# Two-frame apparent motion presented with an inter-stimulus interval reverses optokinetic responses in mice

**DOI:** 10.1038/s41598-018-36260-z

**Published:** 2018-12-13

**Authors:** Kenichiro Miura, Yuko Sugita, Takahisa Furukawa, Kenji Kawano

**Affiliations:** 1 Department of Integrative Brain Science, Graduate School of Medicine, Kyoto University, Kyoto, Japan; 2 Laboratory for Molecular and Developmental Biology, Institute for Protein Research, Osaka University, Osaka, Japan

## Abstract

Two successive image frames presented with a blank inter-stimulus interval (ISI) induce reversals of perceived motion in humans. This illusory effect is a manifestation of the temporal properties of image filters embedded in the visual processing pathway. In the present study, ISI experiments were performed to identify the temporal characteristics of vision underlying optokinetic responses (OKRs) in mice. These responses are thought to be mediated by subcortical visual processing. OKRs of C57BL/6 J mice, induced by a 1/4-wavelength shift of a square-wave grating presented with and without an ISI were recorded. When a 1/4-wavelength shift was presented without, or with shorter ISIs (≤106.7 ms), OKRs were induced in the direction of the shift, with progressively decreasing amplitude as the ISI increased. However, when ISIs were 213.3 ms or longer, OKR direction reversed. Similar dependence on ISIs was also obtained using a sinusoidal grating. We subsequently quantitatively estimated temporal filters based on the ISI effects. We found that filters with biphasic impulse response functions could reproduce the ISI and temporal frequency dependence of the mouse OKR. Comparison with human psychophysics and behaviors suggests that mouse vision has more sluggish response dynamics to light signals than that of humans.

## Introduction

The visual system captures moment-by-moment changes in light signals projected onto the retina to perceive motion in the environment, and drive compensatory behaviors. In general, changes in the projected image are only detectable when the rate of change is within a certain range; too slow or too fast changes are often missed. This limitation is due to temporal filters embedded in visual processing pathways. Previous human psychophysics studies have inferred the impulse response functions of temporal filters underlying visual perception^1,2^.

Temporal filters are crucial elements of visual motion detectors;^3–5^ their characteristics can be revealed by the illusory motion percept^6–9^. When two successive image frames are presented with a blank inter-stimulus interval (ISI), human observers often perceive motion in the opposite direction to actual image shift^6–8,10,11^. In human psychophysics, this directional reversal has been explained by biphasic impulse responses of the temporal filters embedded in the visual motion processing pathway^6–8^.

Motion perception and smooth pursuit responses share visual motion signals^12^; therefore, the characteristics of temporal filters could be revealed by oculomotor responses as well. In humans and non-human primates, motion of a large-field visual pattern induces an ocular following response (OFR), usually in the direction of visual motion^13–16^. This constitutes the initial part of the optokinetic response/reflex (OKR)^17–20^, and shares its visual processing pathway with smooth pursuit eye movement^19^. OFR directional reversal has been demonstrated in humans through presentation of two successive image frames with an ISI^21–23^. Because lesions in the middle temporal and medial superior temporal areas impair OFRs^24^, their directional reversal is thought to be a characteristic of temporal filters underlying cortical visual motion processing.

OKRs are observed in various animal species in addition to humans and non-human primates. Rabbit and rodent OKRs are less influenced by lesions and silencing of the cerebral cortex^25–27^. As such, OKRs of afoveate animals are believed to rely on subcortical neural substrates, such as the retina and brainstem circuitries. The retina is the critical structure for extracting visual motion in mice; direction-selective ganglion cells provide visual motion signals that act downstream to generate OKRs^28,29^. Thus, OKRs in mice may reflect visual processing in the retina. Recent studies have revealed substantial differences between primates and mice in the spatiotemporal-frequency dependence of their OKRs to drifting grating stimuli. The optimal temporal frequency is much lower in mice than in primates^30–32^. Thus, the OKR system in mice may prefer slower changes in luminance patterns than that in humans. This suggests a difference in visual temporal filter characteristics between mice and primates. However, there is currently no finding showing the properties of mouse temporal filters that underlie OKRs.

**Figure 1:**
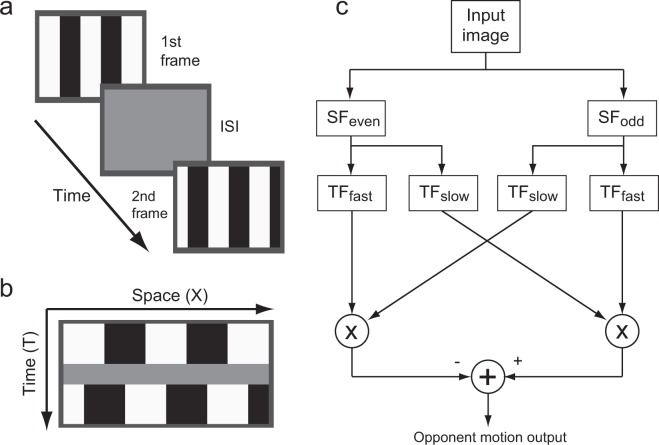
(**a**) Sequence of visual stimuli presented during a trial. (**b**) Schematic diagram of the X-T image, representing a sequence of visual stimuli used as the input image for model analyses. (**c**) Reichardt motion detector used to characterize the effect of ISIs. SF_x_: spatial filters; TF_y_: temporal filters. See text for details.

We herein studied visual temporal filter characteristics underlying mouse OKRs. In this study, OKRs to a 1/4-wavelength shift of gratings presented with ISIs (Fig. 1a,b) were examined to test whether the ISI reverses OKR direction in mice, as it does in motion perception and OFRs in humans. We also quantitatively estimated the temporal filters embedded in mouse vision by using a motion detector model (Fig. 1c). Computer simulations revealed that the estimated filters could successfully reproduce the temporal characteristics of vision underlying OKRs in mice.

## Results

### Effect of ISIs on OKRs

**Figure 2:**
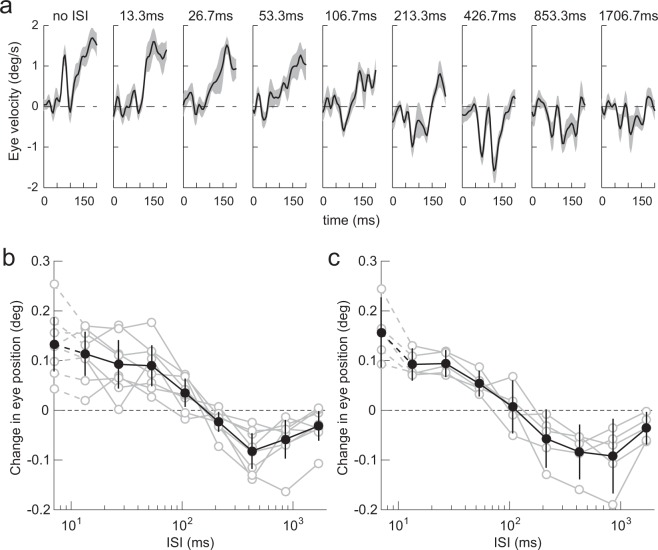
ISI dependence. (**a**) Eye velocity profiles of ocular responses elicited by a 1/4-wavelength step of a square-wave grating (average over eight mice). Upward deflections denote eye movements in the direction of stimulus shift. The gray-hatched areas indicate 95% confidence intervals. The corresponding ISI is indicated at the top of each panel. (**b**) Dependence on ISI quantified from the data shown in (**a**). Data from individual mice (gray open circles, *n* = 8), and their average (black closed circles), are shown. Error bars are 95% confidence intervals. The responses when the ISI was zero are plotted on the vertical axis. Eye movements in the direction of stimulus shift are plotted as positive. Note the logarithmic abscissa. (**c**) Dependence on ISI of initial OKRs elicited by a 1/4-wavelength step of a sinusoidal grating. Other conventions are as in (**b**).

A 1/4-wavelength shift of vertical square-wave grating elicited OKRs in mice. Figure 2a shows the eye velocity profiles of the ocular responses averaged over the eight mice. To quantify the responses, we calculated the change in eye position over a 150-ms interval, starting at 50 ms after stimulus motion onset (Fig. 2b, black closed circles, average over the 8 mice). The direction of the ocular responses was the same as that of the 1/4-wavelength shift when there was no ISI. The responses were statistically significant in this condition (t-test, *t* = 5.75, *p* = 7.01 × 10^−4^). Ocular responses gradually declined as the ISI increased to 106.7 ms. When the ISI was approximately 106.7 ms, ocular responses began to reverse. Ocular responses consistently reversed when the ISI was 213.3 ms or longer (t-test, *t* < −2.74, *p* < 2.89 × 10^−2^), with the largest reversal at 426.7 ms (t-test, *t* = −5.40, *p* = 1.01 × 10^−3^). The reversed response was still significant at 1706.7 ms ISI (t-test, *t* = −2.48, *p* = 4.24 × 10^−2^). Note that similar dependence on ISIs was observed in all mice (Fig. 2b, gray open circles).

The luminance profile of a square-wave grating is mathematically given by summing the odd harmonics with progressively decreasing amplitudes, and a half of the harmonics, i.e., (4*n* − 1)^th^ harmonics (*n*: integer), shifted in the opposite direction when a quarter wavelength step is applied. In an additional experiment, we tested whether higher harmonics are critical for directional reversals using a pure sinusoidal grating. The effects of ISIs obtained from five mice using a sinusoidal grating showed similar characteristics to those elicited by the square-wave grating (Fig. 2c). Thus, the effects of ISIs can be generalized over different grating types; this suggests that directional reversal occurs irrespective of higher harmonics.

### Model analyses

Dependence on ISIs observed using a square-wave grating was analyzed using the motion detector model shown in Fig. 1c. In the model analyses, ocular responses were assumed to be proportional to the opponent motion output of the model. The free parameters of the temporal filters were optimized for each of the eight mice, and also for data averaged over the eight mice under three combinations of filter orders, *N*_*f*_ (=1, 2, 3) and *N*_*s*_ = *N*_*f*_ + 1. The motion detector model could successfully reproduce the dependence on ISIs with appropriate filter parameters under all tested combinations of *N*_*f*_ and *N*_*s*_. Tables 1 and 2 summarize the optimal filter parameters and coefficient of determination, showing the goodness of fit (mean ± standard deviation) obtained from the optimizations performed for each of the eight mice (Tables 1, 1-TS filters; Tables 2, 2-TS filters, see Methods for details).

**Table 1:** Best-fit parameters of the 1-TS filter obtained from individual mice.

	Filter order		
	*N*_*f*_ = 1	*N*_*f*_ = 2	*N*_*f*_ = 3
*k*	7.36 ± 1.21	9.32 ± 1.75	12.62 ± 2.90
*b*	0.75 ± 0.07	0.78 ± 0.07	0.78 ± 0.06
*R* ^2^	0.88 ± 0.11	0.87 ± 0.11	0.85 ± 0.12
AIC	−52.62 ± 10.39	−50.79 ± 9.57	−49.16 ± 8.97
AIC_C_	−42.62 ± 10.39	−40.79 ± 9.57	−39.16 ± 8.97

Mean ± SD of the eight mice. *N*_*s*_ = *N*_*f*_ + 1 for all models.

**Table 2:** Best-fit parameters of the 2-TS filter obtained from individual mice.

	Filter order		
	*N*_*f*_ = 1	*N*_*f*_ = 2	*N*_*f*_ = 3
*k*_*e*_	13.60 ± 4.92	17.59 ± 5.97	21.59 ± 6.26
*k*_*i*_	3.38 ± 1.52	4.26 ± 1.85	4.92 ± 1.76
*b*	0.22 ± 0.21	0.20 ± 0.20	0.16 ± 0.13
*R* ^2^	0.91 ± 0.10	0.91 ± 0.10	0.91 ± 0.10
AIC	−54.76 ± 9.66	−54.65 ± 9.66	−54.50 ± 9.67
AIC_C_	−34.76 ± 9.66	−34.65 ± 9.66	−34.50 ± 9.67

Mean ± SD of the eight mice. *N*_*s*_ = *N*_*f*_ + 1 for all models.

**Figure 3:**
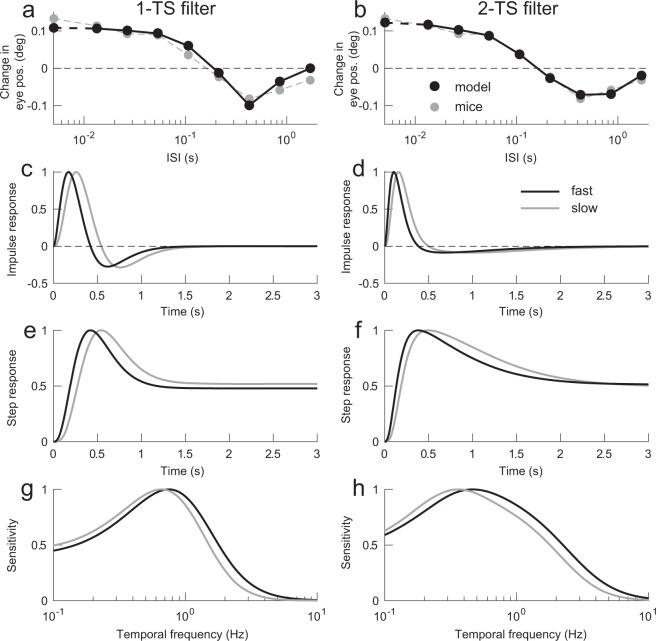
Model analyses. (**a,b**) Comparisons between the mean of the eight mice (gray circles), and simulated responses (black circles) from the best-fit 1-TS (**a**) and 2-TS filter models (**b**). Other conventions are as in Fig. 2. (**c,d**) Impulse response functions (IRFs) of the best-fit 1-TS (**c**) and 2-TS filters (**d**). (**e,f**) Step responses of the best-fit 1-TS (**e**) and 2-TS models (**f**). (**g,h**) Frequency characteristics of the best-fit 1-TS (**g**) and 2-TS filters (**h**). In (**c**–**h**), black and gray traces are fast (*N*_*f*_ = 2), and slow (*N*_*s*_ = 3) filters, respectively. The traces are normalized using their maximal values.

The motion detector models successfully fit the mean responses of the eight mice. Figure 3a,b compare the average of the eight mice with the outputs of the two motion detector models when *N*_*f*_ = 2, and *N*_*s*_ = 3 (a: 1-TS filter, b: 2-TS filter). Both motion detector models achieved good approximations of ISI dependence (*R*^2^ = 0.95, AIC = −54.7, AIC_C_ = −44.7 for 1-TS filter; *R*^2^ = 0.99, AIC = −66.4, AIC_C_ = −46.4 for 2-TS filter, where *R*^2^ is coefficient determination, AIC is Akaike’s information criterion and AIC_C_ is AIC with small sample correction). Differences between the two models’ outputs were seen primarily at the longest ISIs. In this case, the 2-TS filter model, which is obtained from the mean responses of the eight mice, showed better fit compared with the 1-TS filter model. However, the 2-TS filter models obtained from the individual mice had larger AIC_C_ than the 1-TS filter model on average, although they showed better approximations in terms of coefficient of determination (see Tables 1 and 2).

Figure 3c,d show the impulse response functions of the fast (black line), and slow (gray line) temporal filters that achieved the approximations shown in Fig. 3a,b, respectively. The best-fit impulse response functions were similarly characterized as positive and negative lobes, i.e. biphasic, independent of filter type. Note that the negative lobe of 2-TS filters was more elongated than the positive lobe. This was because the estimated *k*_*i*_ (time scale of the negative component) was smaller than *k*_*e*_ (time scale of the positive component) (t-test, *t* = 6.03, *p* = 3.05 × 10^−5^, see Table 2). The step responses of the fast and slow filters are shown in Fig. 3e,f. Both models’ step responses showed transient increases followed by sustained responses of approximately 50% of the peaks. The Fourier transform of the temporal filters indicated band-pass characteristics broadly tuned with center frequencies of 0.75 Hz and 0.66 Hz, for the fast and slow filters, respectively, of the 1-TS filter model. Center frequencies of the 2-TS filter model were 0.47 Hz and 0.36 Hz, for the fast and slow filters, respectively (Fig. 3g,h).

### Model simulations

Computer simulations were performed to examine the mechanisms underlying the ISI effects, and related properties of the initial OKRs. In the following computer simulations, the best-fit model, involving the 2-TS filters (*N*_*f*_ = 2 and *N*_*s*_ = 3, *k*_*e*_ = 18.95, *b* = 0.09, *k*_*i*_ = 3.06, Fig. 3d), was used.

**Figure 4:**
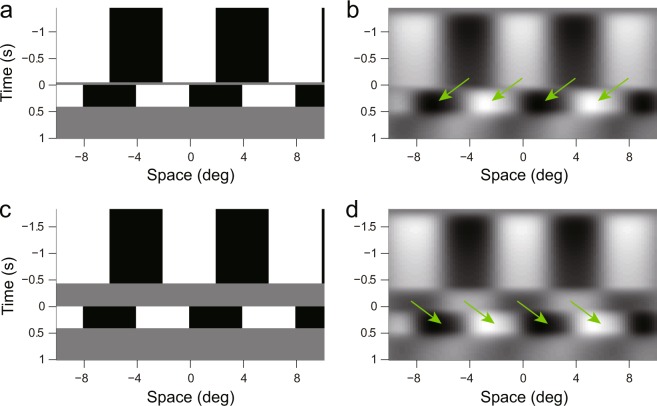
Output of a spatiotemporal separable filter. (**a,c**) are input images in X-T format with ISIs of 0.05 and 0.43 s, respectively; (**b,d**) are filter outputs. Arrows indicate shifts of the patterns around time zero. Time zero indicates when the second frame was presented.

Figure 4 shows the output of a spatiotemporal separable filter under two ISI conditions, to visualize the effects of ISIs. The filter had a Gabor spatial profile (even Gabor function, center frequency: 0.125 cycles/°; spatial constant: 3.0 °), and a biphasic temporal profile (fast filter shown in Fig. 3d). With 53.3 ms ISI (Fig. 4a,b), the output of the spatiotemporal filter during the ISI had the same phase as the first frame; the second frame was presented at this time (*t* = 0). We could see a shift of luminance pattern in the direction of the stimulus step around *t* = 0 (indicated by arrows). On the other hand, when the ISI was 426.7 ms (Fig. 4c,d), the output of the spatiotemporal filter had the opposite sign to the input image during the ISI, and when the second frame was presented (*t* = 0), we could see a luminance pattern shift in the opposite direction of the actual stimulus shift around time zero. These results suggest the mechanism by which directional reversal occurs in mouse OKRs.

**Figure 5:**
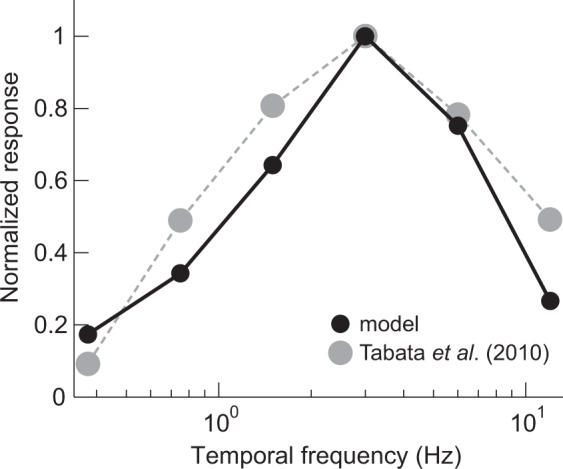
Temporal frequency tuning. Outputs of the best-fit model with 2-TS filters (black circles, solid line) are plotted against the temporal frequencies of the drifting sinusoidal gratings. The OKR amplitudes averaged from three C57BL/6 J mice, replotted from Tabata *et al*.^30^ (gray circles), are superimposed.

Initial OKRs to drifting sinusoidal gratings showed a band-pass temporal frequency tuning that peaked at 3.0 Hz in C57BL/6 J mice^30^. Computer simulations were carried out using the six X-T images of a sinusoidal grating with 0.125 cycles/°, each of which simulated motion at one of six temporal frequencies (0.375, 0.75, 1.5, 3, 6, or 12 Hz). The model outputs, which were normalized so that the maximal output was 1, showed band-pass characteristics and peaked at 3 Hz (Fig. 5, black circles). We replotted our previous C57BL/6 J mouse data (averaged over three mice) from Tabata *et al*.^30^ (Fig. 5, gray circles). The characteristics of the best-fit model were similar to the temporal frequency tuning of the mice in the previous study.

## Discussion

Previous studies have demonstrated that perceived direction of motion reverses when a two-frame apparent motion stimulus is presented with an ISI in humans^6–8,10,11^. Reversed motion percepts were induced even with shorter ISIs (30–70 ms). With ISIs longer than 100 ms, the perceived direction of motion becomes veridical or has no clear directional bias. A previous study demonstrated that motion perception shares visual motion signals with ocular tracking^12^; similar effects of ISIs were observed in human OFRs^21–23^. OFRs to a 1/4-wavelength shift of sinusoidal gratings were elicited in the opposite direction to the actual shift when presented with a 10 ms ISI. The amplitude of the reversed ocular responses peaked at 20–40 ms ISI, and decreased to almost zero with a 100 ms or longer ISI. In this study, we demonstrated that ocular responses in mice also reversed when an ISI was introduced (Fig. 2). However, mouse dependence on ISIs was different to that in perceived motion and OFRs in humans. In mice, the direction of ocular responses reversed for longer ISIs (>213.3 ms), peaking at 426.7 ms.

Previous studies explained ISI effects using the assumption that motion detectors receive signals mediated by temporal filters with biphasic impulse responses^6,8^. The reversed motion percept associated with ISIs was simulated using the motion energy model^3^, which assumes that the temporal filters are biphasic^7,9,33^. Similar arguments have been made regarding the effects of ISIs on OFRs^21–23^. Several previous studies have quantitatively demonstrated temporal filters using flicker-fusion, double-pulse presentations, and two-stroke apparent motion paradigms^1,2,34,35^, as well as two-frame apparent motion stimuli presented with ISIs^23^. Notably, Ohnishi *et al*.^23^ demonstrated that biphasic temporal filters with a zero crossing at ~60 ms best explained the effects of ISIs on human OFRs. As in previous studies, we found that temporal filters with biphasic impulse responses explained the ISI effects. However, the temporal filters that best explained the mouse data showed biphasic impulse response functions with a zero crossing at >400 ms (Fig. 3c,d). These results suggest that the temporal dynamics of the mouse visual system are several times slower than those of humans.

The present study also demonstrated that the motion detector model could reproduce the optimal temporal frequency of drifting sinusoidal gratings for initial OKRs. The model outputs were maximal when temporal frequency of the drifting grating was 3 Hz. This temporal frequency tuning is quite similar to our previous findings^30–32^. In human and non-human primate OFRs, the optimal temporal frequency of drifting sinusoidal gratings was estimated to be 10–20 Hz^13,15,36,37^, which is much higher than the initial OKRs of mice. The differences in temporal frequency tunings between primates and mice can be explained by slower temporal dynamics of the mouse visual system.

The difference in temporal dynamics between the perception/behaviors of humans, and initial OKRs in mice, might be due to their neural substrates. Perceived motion and OFRs in humans and non-human primates are mediated by cortical structures^24,37–42^. In contrast, the OKRs of mice are thought to be governed by subcortical structures^25–27^. In mouse OKRs, the retina is a crucial structure, in which visual motion is detected by direction-selective ganglion cells^28,29^. These cells then provide inputs to the brainstem nuclei responsible for OKRs, involving the nucleus of the optic tract, and the accessory optic system^43–50^. Thus, the temporal filters estimated here might reveal the temporal characteristics of retinal processing to extract visual motion. Previous studies using electroretinogram have suggested that these temporal filters have low-pass, or band-pass characteristics^51–53^. Pandarinath *et al*.^54^ demonstrated that ON and OFF ganglion cells responded to a broad range of temporal frequencies, from 0.15–6 Hz, with drifting sinusoids. However, currently, no finding is available for ON and ON-OFF direction-selective ganglion cells that should be closely related to the OKRs.

In this study, we used two types of temporal filters with different complexity. The motion detector model with the best-fit 1-TS filter reasonably reproduced the dependence on ISIs. However, the responses at the longest ISI were not reproduced by this simpler filter. The model with the 1-TC filter produced almost zero responses at the longest ISI. On the other hand, the 2-TC model could successfully reproduced the responses even at the longest ISIs. Thus, the model with the 2-TS filter provided better approximations compared with the 1-TS filter based on the *R*^2^ values. In general, the 2-TS filter showed smaller AIC values than the 1-TC filter. However, in terms of AIC_C_ shown in Tables 1 and 2, we could not necessarily conclude that the 2-TS filter is superior to the 1-TC filter.

In the present study, we demonstrated that a version of the Reichardt model could explain the characteristics of visual motion processing underlying OKRs in mice. Comparisons between the computational elements of motion detectors, and the neural substrates/activities of the retina might reveal how motion detectors are implemented into biological structures. Studies of this nature are difficult in primates because primate visual motion analyses are performed in the visual cortex. Temporal filters, as well as spatial filters, are crucial elements of motion detection. An exposure to the light signals more or less causes desensitization of photoreceptors depending on strength and exposure duration of light signals. The bleaching of pigments is known to be related to this phenomenon. Such a photoreceptor bleaching might also be related to the ISI effects observed here. The combined effect of a photoreceptor bleaching and a mono-phasic low-pass filter might act as a biphasic temporal filter and produce an afterimage during a blank ISI as shown in Fig. 4d. Other possible causes of the biphasic nature of temporal filters might involve the properties of signal transmissions between cells, membrane properties of cells and characteristics of neural circuits. The filter we described here is an approximation of the entire retinal process that involves multiple biological mechanisms described above. At present, we cannot specify the exact mechanisms underlying these temporal filters because the model itself does not have enough resolution to resolve this problem. Future studies to elucidate the biological mechanisms underlying these filters are necessary. This will considerably progress our knowledge of visual motion processing in the retina.

## Methods

Most of the techniques used for animal preparation, eye movement recording, and visual stimulation were similar to those described in our laboratory’s previous studies^30–32,55,56^.

### Animal preparation

Data were collected from thirteen C57BL/6 J male mice that weighed 19.8–25.5 g (3–4 months old). A head holder was surgically implanted into each mouse that stereotactically fixed the head during experiments. Mice were anesthetized with an intraperitoneal injection of a mixture of ketamine hydrochloride and xylazine hydrochloride and mounted on the stereotaxic apparatus (Narishige, Tokyo, Japan). After an incision was made to expose the skull surface, the skull position was adjusted so that the bregma-lambda axis was horizontal. The head holder was fixed to the top of the skull using stainless steel screws and dental cement. Before any experiments were performed, the animals were able to fully recover from surgery. During experiments, the animal was restrained by bolting the attached head holder to a rigid rod at the center of a platform. All experiments were performed in accordance with protocols approved by the Animal Care and Use Committee of Kyoto University, Animal Experimental Committees of the Institute for Protein Research at Osaka University (approval ID 29-01-2), and with guidelines laid down by the National Institutes of Health, USA, regarding the care and use of animals for experimental procedures.

### Eye movement recording

The right eye was illuminated by infrared light-emitting diodes, and monitored with a CCD camera. Data were analyzed on a computer (Endeavor, Epson, Nagano, Japan) by using image-processing software (Geteye, Matsuura-Denko-sha, Kanazawa, Japan) that calculates the center of the pupil, and measures its position using the subpixel resolution (0.33 °/pixel) at intervals of 5 ms^30^. Recordings were performed in darkness.

### Visual stimulation and procedures

Visual stimuli were presented on three 19-inch monitors (1280 × 1024 pixels, 75 Hz refresh rate, LCD, Mitsubishi, Tokyo, Japan) that were placed in front, and at both sides of the animal, covering 270 ° × 76.6 ° (azimuth × height) of the visual field. This arrangement allowed for binocular stimulation. Each monitor was located at a distance of 19 cm from the center of the platform on which the head of the mouse was fixed. The eyes of the mouse were positioned 13 cm above the platform. Visual stimuli were generated and presented using MATLAB (Mathworks, Natick, MA, USA), and Psychophysics Toolbox extensions^57^.

We examined the effect of ISIs on the initial phase of the OKR. The visual stimulus was a two-frame animation with a 1/4-wavelength shift of a vertical square wave grating (spatial frequency: 0.125 cycles/°; Mickelson contrast: 96%; mean luminance: 100 cd/m^2^), presented with an ISI (Fig. 1a). We used a high contrast stimulus to increase the signal-to-noise ratio of the eye movement signals since the initial OKR magnitude of mice monotonically increases as contrast increases^30^. The ISI of each trial was selected randomly from the following lookup table: 0, 13.3, 26.7, 53.3, 106.7, 213.3, 426.7, 853.3, or 1706.7 ms. The shift direction was either clockwise or counter-clockwise. There were at least 10 trials for each stimulus condition, for each mouse. The initial, and second image patterns were presented for 1400 ms, and 400 ms, respectively. We set the duration of the initial image pattern to be slightly longer than those adopted for previous ISI experiments in humans (≤1000 ms)^6,8,21–23^, so that the ISI was introduced after the visual system reached steady state for the first frame. Note that mouse temporal resolution was expected to be lower than that in humans, as described in the Introduction. A gray background of the mean luminance was presented for 1 s between trials. We used eight mice in this experiment. We also performed an additional experiment using a sinusoidal grating (spatial frequency: 0.125 cycles/°; Mickelson contrast: 32%; mean luminance: 100 cd/m^2^) on five mice to see the generality of the findings.

### Data analyses

Eye-position data were smoothed with a four-pole digital Butterworth filter (−3 dB at 20 Hz); velocity and acceleration traces were derived from a two-point difference algorithm. Trials with saccadic movements (eye velocity >30 °/s, eye acceleration >1000 °/s^2^) during the 250-ms interval starting 50 ms before visual motion onset were discarded. The change in eye position during the 150-ms interval starting 50 ms after visual motion onset was calculated for each trial to quantify the responses. To improve the signal to noise ratio, the mean response to clockwise motion was subtracted from the mean response to counter-clockwise motion of the same visual stimulus for each mouse. Since temporal-nasal eye movements were positive in our sign convention, the differential responses were positive when the OKR was in the direction of the stimulus motion. Note that binocular stimulations were used, and systematic directional asymmetry was not considered.

### Model analyses

A version of the Reichardt motion detector formulated by Adelson and Bergen^3^ was used to characterize the influence of ISIs (Fig. 1c). This Reichardt model had a minimal structure that involved even and odd Gabor spatial filters, slow and fast temporal filters, two direction-selective nodes, and circuitry to compute the opponent motion output to capture the essential features of visual motion processing. We adopted the Reichardt motion detector because it fits structurally with the retinal motion detectors. Briefly, the input image was convolved with spatial and temporal filters. The two signals passed through different spatial and temporal filters, and were multiplied to generate directional signals. The directional signals were then compared to obtain the opponent motion output. Although the structure is different, this version of the Reichardt motion detector is formally equivalent to the motion energy model^3^.

We tried two types of filter model to reproduce ISI dependence. One temporal filter type is standard, and is expressed as:

1 $$y(t)={(kt)}^{N}{e}^{(-kt)}(\frac{1}{N!}-b\frac{{(kt)}^{2}}{(N+2)!}),$$

where *k*, *b* and *N* represent a temporal scale (TS), the strength of the negative lobe, and the order, respectively. We called this filter the “1-TS filter.” Additionally, we used another form of temporal filter that had two separate TSs for the positive and negative components (*k*_*e*_ and *k*_*i*_); this is expressed as in eq. 2:

2 $$y(t)=\frac{1}{N!}\{{({k}_{e}t)}^{N}{e}^{-{k}_{e}t}-b{({k}_{i}t)}^{N}{e}^{-{k}_{i}t}\}\,\,.$$

In this model, the delay of the negative component relative to the positive component is represented by the difference between *k*_*e*_ and *k*_*i*_. *N* and *b* determine the strength of the negative lobe and the order, respectively. We called this filter the “2-TS filter.” The slow and fast temporal filters, which had different *N’*s (*N*_*f*_ and *N*_*s*_), were used in the same manner as in the Reichardt detector model^3^. A single spatial channel that consisted of even and odd Gabor filters was used. The center spatial frequency, and spatial constant of these filters were set at 0.125 cycles/°, and 1.5 °, respectively. These parameters were chosen so that the model could approximate the spatial frequency characteristics of the mouse OKR^30–32,55^.

The four spatiotemporal separable filters were created with 2 × 2 combinations of two spatial (even or odd) and two temporal filters (slow or fast). The X-T image, which is a two-dimensional image simulating horizontal (X) and temporal (T) luminance profiles of the visual stimulus, is shown schematically in Fig. 1b. This was generated for each ISI according to the experimental conditions; the luminance profile of the square wave grating was expressed as −1 (black) or 1 (white), with a frequency of 0.125 cycles/° (sampled at 0.2 ° and 0.01 s, respectively). The input images were filtered with each of the four spatiotemporally separable filters. The outputs of two of the four separable filters were multiplied to obtain two directional signals (one for leftward, the other for rightward), which were then subtracted one from the other to calculate the opponent motion output. The opponent motion output was averaged over the 150-ms interval starting from onset of the second frame, and compared with the ocular responses. The filter parameters (*k* and *b* for the 1-TS model, or *k*_*e*_, *k*_*i*_, and *b* for the 2-TS model), and the scale factor (*c’s*) were optimized, so that the sum of squared errors between the predicted and actual responses were minimized for the various combinations of *N*_*f*_ (=1, 2, 3) and *N*_*s*_ (=*N*_*f*_ + 1). We calculated *R*^2^ and Akaike information criterion with and without small sample correction^58^.

## Data Availability

The datasets generated and analyzed during the current study are available from the corresponding author on reasonable request.
